# Delayed‐Onset Hemolysis in a Case of Hemolytic Uremic Syndrome: A Diagnostic Challenge

**DOI:** 10.1155/crh/2941212

**Published:** 2026-02-02

**Authors:** Muhammad Younas, Noor Fatima, Zimal Naveed, Syeda Nafisa

**Affiliations:** ^1^ Acute Internal Medicine Institution: George Eliot Hospital NHS Trust, Nuneaton, Warwickshire, UK

**Keywords:** acute kidney injury, adult case report, delayed hemolysis, hemolytic uremic syndrome, thrombotic microangiopathy

## Abstract

**Background:**

Hemolytic uremic syndrome is a rare thrombotic microangiopathy characterized by hemolytic anemia, thrombocytopenia, and acute kidney injury. While commonly reported in children, adult‐onset presentations are less frequent and often atypical, leading to diagnostic delays. This case underscores the importance of repeated evaluation when classical features are absent initially.

**Case Presentation:**

A 50‐year‐old woman was admitted with diarrhea, vomiting, abdominal pain, and visible hematuria. Initial findings included severe thrombocytopenia and Stage 3 acute kidney injury but no evidence of hemolysis. Blood cultures grew *Escherichia coli* sensitive to piperacillin–tazobactam. Despite intensive care management for septic shock, renal function deteriorated and renal replacement therapy was required. On Day 9 of admission, delayed hemolysis became evident with schistocytes on blood smear, undetectable haptoglobin, and hemoglobin decline from 125 g/L at baseline to 87 g/L. These findings confirmed delayed‐onset hemolytic uremic syndrome. ADAMTS13 activity was not tested because TTP was considered clinically unlikely based on stable coagulation parameters and absence of neurological features. Supportive care, including renal replacement therapy and blood products, was provided, and the patient’s renal function normalized before discharge.

**Conclusion:**

This case highlights the diagnostic complexity of adult‐onset hemolytic uremic syndrome, particularly when hemolysis develops late. Clinicians should maintain a high index of suspicion in adults presenting with unexplained acute kidney injury and thrombocytopenia, even in the absence of early hemolytic markers. Serial blood film reviews and multidisciplinary input are essential to avoid missed or delayed diagnosis. Early recognition enables timely supportive care and consideration of targeted therapies to prevent irreversible renal damage and long‐term complications.

## 1. Introduction

Hemolytic uremic syndrome (HUS) is a thrombotic microangiopathy defined by the triad of microangiopathic hemolytic anemia, thrombocytopenia, and acute kidney injury [1]. Adult‐onset HUS is uncommon and often presents atypically, which can delay diagnosis and management. Population‐based estimates suggest an annual incidence of approximately 0.6 per 100,000 persons overall, with cases more common in children than adults [2]. Adult presentations may lack the complete clinical triad at onset, particularly when sepsis or systemic inflammatory responses coexist, necessitating serial reassessment [1]. This report links these epidemiologic and diagnostic considerations to a case in which hemolysis manifested 9 days into admission, prompting re‐evaluation and confirmation of HUS.

## 2. Case Presentation

A 50‐year‐old woman presented with a 2 day history of profuse diarrhea, vomiting, abdominal pain, visible hematuria, and scleral icterus. She denied recent travel or undercooked meat consumption. On examination, she was febrile (38.9°C), hypotensive (87/55 mmHg), tachycardic (HR 110 bpm), and dehydrated. Abdominal examination revealed left renal angle tenderness. Neurologic examination was normal (no confusion, focal deficits, and seizures), and there were no petechiae, purpura, or rash.

**Figure 1:**
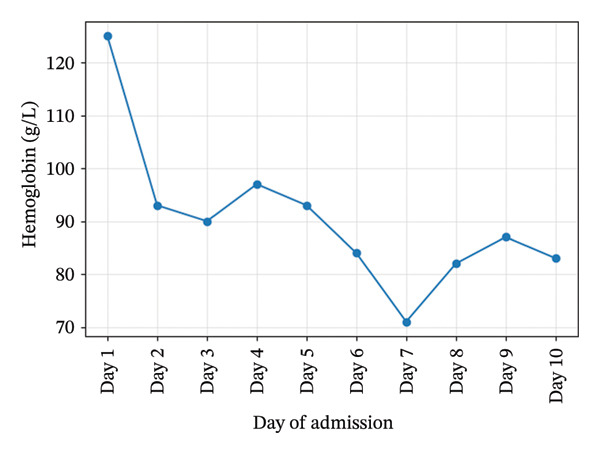
Hemoglobin trends during admission.

**Figure 2:**
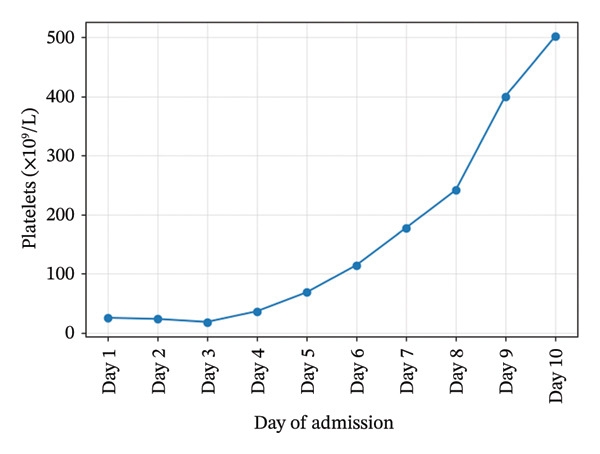
Platelets trends during admission.

**Figure 3:**
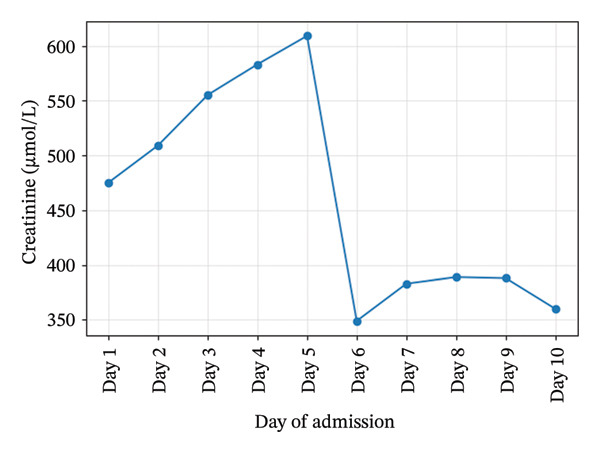
Creatinine trends during admission.

**Figure 4:**
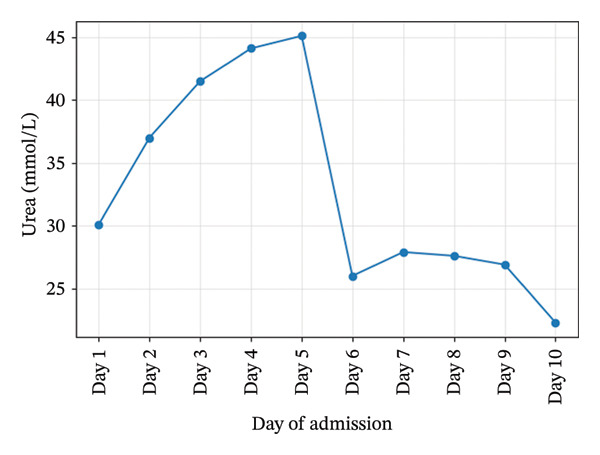
Urea trends during admission.

Initial investigations showed severe thrombocytopenia (platelets 25 × 10^9^/L), Stage 3 acute kidney injury (creatinine 475 μmol/L), leukocytosis (WBC 35.5×10^9^/L), and cholestatic liver enzymes. Hemoglobin, platelet, creatinine, and urea trends during admission are illustrated in Figures 1, 2, 3, and 4. Peripheral smear showed no schistocytes; LDH was raised and haptoglobin was within the reference ranges at admission. Stool culture was negative for Shiga toxin–producing *Escherichia coli* (STEC); blood cultures grew *Escherichia coli* sensitive to piperacillin–tazobactam. Coagulation studies were normal (PT/aPTT), with fibrinogen elevated (6.3 g/L). Complement C3 and C4 concentrations were within normal limits. Laboratory trends during admission are summarized in Table 1.

**Table 1:** Laboratory trends during admission.

Parameter	Day 1	Day 4	Day 9	Day 10
Hemoglobin (g/L)	125	97	87	83
Platelets (x10^9^/L)	25	36	400	501
Creatinine (μmol/L)	475	583	388	360
Urea (mmol/L)	30.1	44.1	26.9	22.3
LDH (U/L)	1523	—	—	—
Haptoglobin (g/L)	0.1	—	—	—
Fibrinogen (g/L)	6.3	—	—	—
Complement C3 (g/L)	1.72	—	—	—
Complement C4 (g/L)	0.55	—	—	—
WBC (x10^9^/L)	35.55	28.44	16.94	12.95

The patient was managed in the intensive care unit for septic shock and multiorgan dysfunction. Despite hemodynamic stabilization, she became anuric and required intermittent hemodialysis. On Day 9 of admission, a repeat peripheral smear revealed schistocytes. Haptoglobin became undetectable, and hemoglobin fell from a baseline of 125 g/L to 87 g/L, confirming delayed‐onset hemolysis and leading to a diagnosis of HUS. Peripheral smear findings are detailed in Table 2.

**Table 2:** Peripheral blood film review.

Day	Findings
Day 1	Genuine thrombocytopenia; left‐shifted neutrophilia; occasional myelocytes.
Day 4	Neutrophilia and mild monocytosis; thrombocytopenia; no clumps.
Day 9	Moderate anisocytosis; mild rouleaux; toxic neutrophils; occasional fragments/schistocytes; large platelet clumps.

ADAMTS13 activity was not tested because thrombotic thrombocytopenic purpura (TTP) was considered clinically unlikely based on stable coagulation parameters and absence of neurological features. Plasma exchange was not initiated for the same reason. Supportive care included intravenous fluids, renal replacement therapy, and red blood cell transfusions. Renal function subsequently improved, urine output recovered, and dialysis was discontinued prior to discharge.

## 3. Discussion

This case illustrates a diagnostically challenging adult‐onset presentation of HUS in which the classic hemolytic markers were absent initially and appeared on Day 9. In adults, distinguishing infection‐associated HUS from complement‐mediated HUS (aHUS) and TTP requires a structured workup, hemolysis markers, peripheral smear for schistocytes, coagulation profile to exclude disseminated intravascular coagulation, and early ADAMTS13 activity to rule in/out TTP [1, 2]. In our case, ADAMTS13 was not tested because TTP was considered clinically unlikely based on stable coagulation parameters and absence of neurological features.

When Shiga toxin–mediated HUS is suspected, stool testing that includes PCR for stx1/stx2 genes is more sensitive than culture alone and is recommended where available [3, 4]. In our patient, stool culture was negative for STEC, and PCR was not performed due to local resource limitations. Complement levels were normal; however, normal complement does not exclude aHUS, and clinical judgment plus ADAMTS13 testing and ongoing reassessment remain essential [5, 6].

Treatment of infection‐associated HUS in adults is predominantly supportive—aggressive hydration, blood pressure control, dialytic support, and transfusion as needed; routine plasma exchange is not indicated for typical STEC‐HUS [7]. By contrast, complement blockade (eculizumab or ravulizumab) is the cornerstone of therapy for aHUS once TTP has been excluded, ideally initiated early to prevent irreversible renal injury [6]. Venous thrombotic events can complicate the convalescent phase of thrombotic microangiopathies due to endothelial injury and a prothrombotic milieu; recognition and appropriate prophylaxis or treatment should be considered on a case‐by‐case basis [8].

Long‐term outcomes after HUS vary: while many patients recover, persistent proteinuria, hypertension, or chronic kidney disease can occur, particularly after prolonged anuria or dialysis [9]. Structured follow‐up is advisable to monitor renal and cardiovascular sequelae. Nonclassical adult presentations, including “partial” HUS with an incomplete triad at onset or delayed hemolysis after gastrointestinal symptoms, are increasingly recognized and underscore the need for serial blood film review and a high index of suspicion [10, 11]. Peripheral smear findings in our case (Table 2) highlight the importance of repeated evaluation when initial results are inconclusive.

## 4. Conclusion

Adult‐onset HUS should remain in the differential for patients presenting with acute kidney injury and thrombocytopenia even when early hemolytic markers are absent. Serial reassessment including repeat blood films and multidisciplinary input are the key to timely diagnosis and appropriate supportive or targeted therapy. Future research should focus on optimal diagnostic pathways in adults with atypical or delayed presentations and on strategies to reduce long‐term renal morbidity.

## Author Contributions

Muhammad Younas, Noor Fatima, and Zimal Naveed drafted the manuscript. Syeda Nafisa reviewed and provided critical feedback.

## Funding

This study received no external funding.

## Disclosure

All authors approved the final version.

## Consent

Written informed consent was obtained from the patient for publication of this case report.

## Conflicts of Interest

The authors declare no conflicts of interest.

## Data Availability

No datasets were generated or analyzed for this case report.
